# Emergency department outcomes for patients experiencing homelessness in England: retrospective cross-sectional study

**DOI:** 10.1093/eurpub/ckac191

**Published:** 2023-01-09

**Authors:** Charlie Moss, Laura Anselmi, Matt Sutton

**Affiliations:** Health Organisation, Policy and Economics (HOPE), Centre for Primary Care and Health Services Research, The University of Manchester, Manchester, UK; Health Organisation, Policy and Economics (HOPE), Centre for Primary Care and Health Services Research, The University of Manchester, Manchester, UK; Health Organisation, Policy and Economics (HOPE), Centre for Primary Care and Health Services Research, The University of Manchester, Manchester, UK

## Abstract

**Background:**

Emergency departments (EDs) are an important point of access to health care for people experiencing homelessness. Evidence suggests that ED attendances by homeless people are more likely to result in leaving the ED without treatment, or dying in the ED. We investigate which diagnoses and patterns of health care use are associated with these (and other) discharge destinations and re-attendance within 7 days among homeless patients.

**Methods:**

We used national hospital data to analyze attendances of all 109 254 people experiencing homelessness who presented at any Type 1 ED in England over 2013–18. We used logistic regression to estimate the association of each outcome with primary diagnosis and patterns of healthcare use.

**Results:**

Compared with patients with no past ED use, patients with a high frequency of past ED use were more likely to leave without treatment and re-attend within 7 days. Patients not registered at a general practice were likelier to leave without treatment or die in the ED and had lower odds of unplanned re-attendance. A primary diagnosis of ‘social problems’ was associated with being discharged without follow-up. Patients with a psychiatric primary diagnosis were disproportionately likely to be referred to another health care professional/provider or an outpatient clinic.

**Conclusions:**

Further research is needed to understand why some homeless patients leave the ED without treatment and whether their healthcare needs are being met. Some patients may be attending the ED frequently due to poor access to other services, such as primary care and social welfare.

## Introduction

The European Typology of Homelessness and Housing Exclusion (ETHOS) sets out a framework for describing different types of homelessness.^1^ They include rooflessness, houselessness, living in insecure housing and living in inadequate housing. A cross-national survey of eight European nations in 2019 found that 4.96% of respondents had experienced rooflessness at some point in their lifetime.^2^

The legal definition of homelessness in England is that a household has no home in the UK or anywhere else in the world available and reasonable to occupy.^3^ This includes several different circumstances, such as sleeping rough, living in a hostel, living in insecure or inadequate housing and sofa surfing. Between April 2020 and March 21, 282 000 single people, couples and families were judged as homeless or threatened with homelessness by local authorities in England.^4^

People experiencing homelessness (PEH) commonly suffer poor physical and/or mental health.^5–8^ Comorbid physical, mental and substance abuse problems are common.^9^ Studies have tended to find a standardized mortality ratio of 2–5 times that of the age-standardized general population.^8^ Deaths of PEH are often from causes amenable to timely and effective health care.^10^

Studies from several high-income countries suggest that, on average, PEH are frequent emergency department (ED) users.^7^^,^^11–17^ This could be due partly to problems in accessing primary health care.^18^^,^^19^ A study of PEH in England found that only 66% of rough sleepers, 83% of single homeless people in accommodation and 89% of hidden homeless people (e.g. sofa surfers) were registered with a general practice, compared with 98% of the English general population.^20^ The way in which EDs operate may be more acceptable to some PEH than the shorter opening hours and rigid appointment schedules at most primary healthcare providers. A substantial portion of this use of emergency health care is warranted by urgent medical need.^20–22^

Much attention has been given to the frequency with which PEH attend the ED.^15^^,^^23^ There has been scant investigation of the outcomes of ED attendances by PEH, particularly outside the USA, even though this may be related to frequent ED attendance by some PEH. A recent study of 3271 ED attendances by PEH at one hospital in England found that 18.4% of patients left the ED before receiving treatment, and 1.2% of patients died in the ED.^24^ Both percentages are significantly higher than for the general population.^25^ A comparative study of ED attendances at a hospital in Dublin, Ireland, found that 40% of attendances by PEH resulted in leaving the ED before assessment, compared with 15% of attendances by housed persons.^14^

We use national hospital data to investigate which diagnoses and patterns of healthcare use are associated with outcomes of the ED attendances of 109 254 homeless patients in England. We examine discharge destinations and unplanned ED re-attendance within 7 days.

## Methods

### EDs in England

In England, health care provided by National Health Service (NHS) EDs is free at the point of use, including to overseas visitors.^26^ It can be routinely accessed without an appointment.

There are four types of ED that patients can choose to attend.^27^^,^^28^ Type 1 are major NHS consultant-led 24-h departments with full facilities for resuscitating patients. They comprise the majority of ED attendances in England. We analyze attendances at Type 1 EDs only.

### Hospital records

Hospital Episode Statistics is an administrative dataset containing information on NHS hospital service use for all ED attendances, admissions and outpatient appointments.^29^ Each patient has a unique anonymized identifier allowing individuals’ records to be linked when they visit different hospital settings and over time. The ED data contain information on diagnoses and treatment, where patients are referred or admitted, discharge destination and some provider and patient characteristics.^28^

### Sample definition

Within the hospital records there are fields derived from the patient’s postcode that assign patients to different administrative geographies. One such field is ‘local authority district of residence’ where patients can be recorded as having ‘no fixed abode’. This is an indicator of homelessness.^7^^,^^21^^,^^30^

We included individuals recorded as having ‘no fixed abode’ at an ED attendance between 1 April 2013 and 31 March 2018. We limited the sample to patients aged 16–75.^21^

For patients with multiple attendances, we kept only their earliest attendance where they were recorded as having no fixed abode to avoid giving greater weight to patients who attended more frequently. We removed 278 records with the same date and arrival time, but conflicting information in other fields. We removed 20 patients who were dead on arrival at the ED. This resulted in a sample of 109 254 PEH (Supplementary figure S1).

### Outcomes

#### Discharge destination

We created binary indicators for each of the following seven possible mutually exclusive outcomes: (i) admitted to hospital; (ii) discharged with no follow-up; (iii) discharged with GP follow-up; (iv) referred to outpatient clinic; (v) referred to other health care professional or provider; (vi) left without treatment (including patients who either left the department before treatment or refused treatment); and (vii) died in the department.

#### Re-attendance within 7 days

Unplanned ED re-attendance within 7 days is an indicator of poor quality care.^25^ We created a binary indicator for whether there was an unplanned follow-up attendance within 7 days of the initial attendance.

### Patient characteristics

We created indicators for 5-year age bands.

We dropped a small number of observations with missing data for sex (1083; 1% of sample).

We grouped ethnic categories into six groups: White British or Irish, any other White background, Mixed, Asian, Black, or other (see Supplementary table S1 for original categories). We retained ‘White from any other background’ as a separate group from White British or Irish to investigate differences. We retained observations for patients with missing ethnicity information, 24% of the sample, and categorized them as ‘missing’.

### Diagnoses

Where present, diagnosis codes are a mixture of NHS Accident and Emergency (A&E) diagnosis condition codes (71 774 attendances; 66% of total)^31^ and ICD-10 codes (4243 attendances; 4% of total).^32^ The first two characters of A&E diagnosis codes correspond to 39 broad diagnosis groups reflecting the conditions at presentation, such as ‘head injury’, ‘cardiac conditions’ and ‘respiratory conditions’ (Supplementary table S2). There were 70 708 attendances (65% of total attendances) that had a valid A&E diagnosis code. For 1066 attendances (1% of total), the first two characters of the A&E diagnosis code were ambiguous. In most cases, this is because they were not in the valid range of 01–39. Thirty percent of the sample (33 237 attendances) did not have a primary diagnosis recorded. We retained observations with an ambiguous or absent code and categorized them as missing. We also categorized the few ICD-10 diagnosis codes as missing, limiting analysis of diagnosis codes to NHS ED codes.

We mapped valid A&E diagnosis codes into one of the following categories: traumatic injury; physical conditions/diseases; poisoning (incl. overdose); psychiatric conditions; social problems; diagnosis not classifiable; and nothing abnormal detected (see Supplementary table S2). We classified poisoning as a separate category because drug use is prevalent amongst PEH and poisoning is one of the most common primary diagnoses received by this population at the ED.^21^

### General practice registration

We used general practice registration status as an indicator of access to primary care.

The ED data contain information on which general practice the patient is registered with. There are three codes which indicate that a patient does not have a registered general practice to record.^33^ We used these codes to identify patients not registered at a general practice. We created an indicator equal to one if any of these codes were present (Supplementary table S3).

### Past healthcare utilization

We used the unique person identifiers to extract data on all hospital interactions across all settings (inpatient, outpatient and ED) for the 5 years prior to each patient’s attendance. From total counts of ED attendances, inpatient spells, and outpatient appointments during the 5 years prior to each patient’s ED attendance, we created 12 binary indicators: zero use; low use (between one and the 40th percentile); moderate use (between 40th and 80th percentile); or high use (above 80th percentile) of ED, inpatient and outpatient care. See Supplementary tables S4 and S5.

### Analyses

We used logistic regression to estimate the association between each outcome and the set of independent variables of interest (diagnosis category; general practice registration status; and frequency of past ED attendances, inpatient spells and outpatient appointments). We additionally included the following binary indicators as control variables in all models: 5-year age bands; sex; ethnic group; and whether the attendance was a first attendance or a follow-up.

We used Huber–White robust standard errors.

## Results

### Descriptive statistics

Twenty-two percent of the sample were female (table 1). The age distribution was skewed to the right; 72% of the sample were in the bottom half of the age range (aged 16–45).

**Table 1:** Descriptive statistics: patient characteristics

	%
Sex	
Female	22.1
Age	
16–20	7.8
21–25	13.2
26–30	14.4
31–35	13.5
36–40	12.3
41–45	11.0
46–50	9.9
51–55	7.2
56–60	4.6
61–65	2.9
66–70	2.1
71–75	1.2
Ethnicity	
White British or Irish	45.0
Any other white background	13.5
Mixed	1.3
Asian	3.9
Black	4.2
Other	7.8
Missing	24.2
*N*	109 254

*Data source*: Hospital Episode Statistics Accident and Emergency (A&E), Type 1 A&E attendances by patients with no fixed abode aged 16–75 between 1 April 2013 and 31 March 2018.

Forty-five percent of the sample were White British or Irish. ‘Any other White background’ was the second largest non-missing group (13.5%).

Diagnosis of physical trauma (14.1%) was the most frequent category after missing (35.3%; table 2); 14.7% of valid primary diagnoses were for poisoning, and 9.2% were for psychiatric conditions (Supplementary table S2). Where a diagnosis was recorded, the vast majority were clinical codes; only 3.3% of valid primary diagnoses were for ‘social problems’, which includes homelessness.

**Table 2:** Descriptive statistics: key independent variables

	%
Diagnoses	
Nothing abnormal detected	3.0
Physical trauma	14.1
Physical condition/disease	13.2
Poisoning	9.5
Psychiatric conditions	6.0
Social problems	2.1
Not classifiable	16.8
Missing	35.3
General practice registration	
Not registered	49.2
Frequency of past ED attendances^a^	
No use	57.9
Low (1–4)	18.3
Moderate (5–15)	16.1
High (>15)	7.8
Frequency of past inpatient spells^a^	
No use	68.6
Low (1–2)	14.5
Moderate (3–7)	10.8
High (>7)	6.1
Frequency of past outpatient appointments^a^	
No use	63.4
Low (1–5)	15.1
Moderate (6–20)	14.4
High (>20)	7.1
*N*	109 254

*Data source*: Hospital Episode Statistics Accident and Emergency (A&E), Type 1 A&E attendances by patients with no fixed abode aged 16–75 between 1 April 2013 and 31 March 2018.

a ‘Past’ refers to hospital care contact during the 5 years prior to the patient’s attendance.

**Table 3:** Descriptive statistics: outcomes

	%
Discharge destination	
Admitted	22.38
Discharged with no follow-up	33.19
Discharged with GP follow-up	16.99
Referred to outpatient clinic	2.39
Referred to other professional or provider	3.1
Left without treatment	14.59
Died in the department	0.19
Re-attendance	
Unplanned within 7 days	1.63
*N*	109 254

SD, standard deviation; GP, general practitioner.

*Data source*: Hospital Episode Statistics Accident and Emergency attendances by patients with no fixed abode aged 16–75 between 1 April 2013 and 31 March 2018.

Statistics are based on the estimation samples used for each outcome. Further details on how the estimation samples are derived are provided as Supplementary material. Discharge destination outcomes are calculated from attendances at Type 1 Accident and EDs only.

**Table 4:** Regression results: discharge destinations and re-attendance within 7 days

	Admitted	Discharged with no follow-up	Discharged with GP follow-up	Referred to outpatient clinic	Referred to other professional or provider	Left without treatment	Died in the department	Unplanned re-attendance within 7 days
	(1)	(2)	(3)	(4)	(5)	(6)	(7)	(8)
Diagnoses								
Nothing abnormal detected								
Traumatic injury	1.78***	1.07	1.60***	5.45***	1.24	0.10***		0.98
	[1.56; 2.02]	[1.00; 1.16]	[1.43; 1.78]	[3.44; 8.63]	[0.90; 1.71]	[0.09; 0.12]		[0.71; 1.37]
Physical conditions/diseases	4.33***	0.61***	1.95***	6.83***	1.11	0.11***		0.95
	[3.82; 4.90]	[0.56; 0.66]	[1.75; 2.17]	[4.31; 10.81]	[0.81; 1.54]	[0.10; 0.13]		[0.68; 1.32]
Poisoning	4.45***	1.00	0.98	0.99	0.64*	0.23***		0.68*
	[3.92; 5.06]	[0.93; 1.09]	[0.87; 1.10]	[0.59; 1.67]	[0.44; 0.91]	[0.21; 0.26]		[0.48; 0.98]
Psychiatric conditions	2.78***	0.59***	1.35***	3.73***	8.40***	0.21***		1.38
	[2.43; 3.17]	[0.54; 0.64]	[1.20; 1.52]	[2.31; 6.03]	[6.19; 11.40]	[0.18; 0.24]		[0.98; 1.93]
Social problems	2.79***	1.31***	1.12	1.96*	0.58	0.23***		1.18
	[2.39; 3.26]	[1.18; 1.46]	[0.96; 1.31]	[1.08; 3.58]	[0.34; 1.01]	[0.19; 0.27]		[0.75; 1.84]
Diagnosis not classifiable	2.62***	0.57***	1.19**	3.02***	2.19***	0.92		1.50*
	[2.31; 2.96]	[0.53; 0.61]	[1.07; 1.32]	[1.90; 4.80]	[1.62; 2.98]	[0.84; 1.00]		[1.09; 2.05]
Missing	3.07***	0.50***	1.17**	4.40***	2.91***	0.82***		1.39*
	[2.72; 3.47]	[0.46; 0.53]	[1.05; 1.30]	[2.79; 6.93]	[2.16; 3.93]	[0.75; 0.89]		[1.02; 1.89]
General practice registration								
Not registered	0.96	1.11***	0.85***	0.78***	0.99	1.18***	1.68*	0.41***
	[0.92; 1.00]	[1.07; 1.15]	[0.81; 0.89]	[0.69; 0.87]	[0.89; 1.09]	[1.12; 1.24]	[1.09; 2.57]	[0.31; 0.53]
Frequency of past ED attendances^a^								
No use								
Low	1.16***	0.98	1.00	0.99	0.90	0.87***	1.14	6.03***
	[1.10; 1.24]	[0.93; 1.03]	[0.94; 1.07]	[0.85; 1.16]	[0.78; 1.03]	[0.81; 0.93]	[0.64; 2.02]	[4.45; 8.16]
Moderate	0.98	1.01	0.99	0.94	0.88	1.17***	0.47	6.97***
	[0.91; 1.05]	[0.95; 1.08]	[0.91; 1.06]	[0.78; 1.13]	[0.74; 1.03]	[1.07; 1.27]	[0.20; 1.09]	[5.05; 9.60]
High	0.72***	1.16***	1.10	0.62***	0.62***	1.62***	0.21**	10.79***
	[0.65; 0.78]	[1.07; 1.27]	[0.99; 1.22]	[0.48; 0.81]	[0.49; 0.78]	[1.45; 1.82]	[0.07; 0.65]	[7.68; 15.16]
Frequency of past inpatient spells^a^								
No use								
Low	1.25***	0.89***	0.93*	0.96	1.14	0.99	1.35	1.04
	[1.18; 1.32]	[0.85; 0.94]	[0.87; 0.99]	[0.84; 1.11]	[0.99; 1.30]	[0.92; 1.07]	[0.72; 2.53]	[0.90; 1.21]
Moderate	1.78***	0.78***	0.80***	0.85	1.17	0.92	2.36*	1.07
	[1.66; 1.90]	[0.73; 0.83]	[0.74; 0.86]	[0.71; 1.01]	[0.99; 1.39]	[0.84; 1.00]	[1.08; 5.14]	[0.91; 1.27]
High	2.51***	0.67***	0.65***	1.05	1.30*	0.81***	2.34	1.09
	[2.29; 2.75]	[0.61; 0.73]	[0.58; 0.73]	[0.81; 1.35]	[1.03; 1.65]	[0.72; 0.92]	[0.86; 6.38]	[0.88; 1.34]
Frequency of past outpatient appointments^a^								
No use								
Low	1.09**	0.97	0.99	1.19*	0.98	0.86***	0.64	1.02
	[1.03; 1.16]	[0.93; 1.03]	[0.93; 1.06]	[1.02; 1.38]	[0.85; 1.13]	[0.80; 0.92]	[0.33; 1.21]	[0.86; 1.20]
Moderate	1.08*	0.94*	1.00	1.30**	1.09	0.81***	0.71	1.04
	[1.02; 1.15]	[0.89; 1.00]	[0.93; 1.07]	[1.11; 1.53]	[0.94; 1.27]	[0.75; 0.88]	[0.35; 1.44]	[0.88; 1.24]
High	1.12**	0.96	0.95	1.29*	1.21*	0.77***	1.43	0.94
	[1.03; 1.21]	[0.89; 1.03]	[0.87; 1.04]	[1.05; 1.58]	[1.00; 1.45]	[0.69; 0.85]	[0.69; 2.98]	[0.76; 1.15]
*N*	109 254	109 254	109 254	109 254	109 254	109 254	105 855	109 027

95% confidence intervals in brackets.

*Data source*: Hospital Episode Statistics Accident and Emergency, Type 1 A&E attendances by patients with no fixed abode aged 16–75 between 1 April 2013 and 31 March 2018.

Estimated using logistic regression. Coefficients show odds ratios.

Models additionally include binary indicators for sex, 5-year age band, ethnicity and whether the attendance is categorized as a first attendance, planned follow-up, unplanned follow-up or unknown.

Due to small number of patients who died in the department, diagnosis indicators were not included in Model 7 due to perfect prediction. Also includes first attendances only due to perfect prediction.

Model 8 does not include attendances with attendance type or department type ‘unknown’ due to perfect prediction.

a Past refers to hospital care contact in the 5 years prior to the attendance.

* *P* < 0.05,

** *P* < 0.01,

*** *P* < 0.001.

For 49% of patients, their general practice code indicated that they were not registered with a general practice (table 2).

Twenty-two percent of attendances concluded with admission to hospital, 33% with discharge without follow-up and 15% with the patient leaving without treatment (table 3); 0.2% of patients died in the ED.

Fifty-six percent of patients arrived by ambulance (Supplementary table S6). Around half of patients were self-referred; 19% were referred by emergency services; and 7% were referred by the police.

### Regression results

Compared with the reference category of nothing abnormal detected, all other diagnosis categories had lower odds of leaving without treatment (table 4). The diagnoses with the largest odds ratios were ‘diagnosis not classifiable’ (OR = 0.92; 95% CI [0.84–1.00]) and missing primary diagnosis (OR = 0.82; 95% CI [0.75–0.89]). Patients with a primary diagnosis of social problems were much less likely to leave without treatment (OR = 0.23; 95% CI [0.19–0.27]).

Compared with the reference diagnosis of nothing abnormal detected, patients with poisoning (OR = 4.45; 95% CI [3.92–5.06]) or a physical condition/disease (OR = 4.33; 95% CI [3.82–4.90]) were most likely to be admitted to hospital. Social problems was the only diagnosis where the patient was more likely to be discharged with no follow-up, compared with a diagnosis of nothing abnormal detected (OR = 1.31; 95% CI [1.18–1.46]). Being diagnosed with a psychiatric condition was associated with a greater probability of being referred to another professional/provider (OR = 8.40; 95% CI [6.19–11.40]).

Patients with a primary diagnosis that was not classifiable (OR = 1.50; 95% CI [1.09–2.05]) or missing primary diagnosis (OR = 1.39; 95% CI [1.02–1.89]) were more likely to have an unplanned re-attendance within 7 days.

Patients who were not registered with a general practice were more likely to be discharged with no follow-up (OR = 1.11; 95% CI [1.07–1.15]), leave without treatment (OR = 1.18; 95% CI [1.12–1.24]), or die in the ED (OR = 1.68; 95% CI [1.09–2.57]) compared with patients with a registered general practice recorded. They were less likely to be discharged with GP follow-up (OR = 0.85; 95% CI [0.81–0.89]), be referred to an outpatient clinic (OR = 0.78; 95% CI [0.69–0.87]), or have a re-attendance within 7 days (OR = 0.41; 95% CI [0.31–0.53]).

Patients with high frequency of past ED attendances were more likely to be discharged with no follow-up (OR = 1.16; 95% CI [1.07–1.27]), leave without treatment (OR = 1.62; 95% CI [1.45–1.82]), or have a re-attendance within 7 days (OR = 10.79; 95% CI [7.68–15.16]), compared with patients without prior ED use. They were also less likely to be admitted to hospital (OR = 0.72; 95% CI [0.65–0.78]) or referred to another provider (OR = 0.62; 95% CI [0.49–0.78]) or outpatient clinic (OR = 0.62; 95% CI [0.48–0.81]).

Patients with high frequency of past admission spells were more likely to be admitted to hospital (OR = 2.51; 95% CI [2.29–2.75]) and less likely to leave without treatment (OR = 0.81; 95% CI [0.72–0.92]) than patients with no past admission spells. They were also less likely to be discharged with no follow-up (OR = 0.67; 95% CI [0.61–0.73]) or with GP follow-up (OR = 0.65; 95% CI [0.58–0.73]).

Similarly, patients with high frequency of past outpatient appointments were more likely to be admitted to hospital (OR = 1.12; 95% CI [1.03–1.21]) and less likely to leave without treatment (OR = 0.77; 95% CI [0.69–0.85]), compared with patients with no past outpatient appointments.

## Discussion

We aimed to investigate which diagnoses and patterns of health care use are associated with different ED discharge destinations and probability of re-attendance within 7 days in a sample of 109 254 homeless patients. We found that the outcomes were associated with certain diagnoses and patterns of healthcare use.

Frequent past use of the ED and not being registered with a general practice were factors associated with leaving the ED without treatment. This may be indicative that some patients who were recorded as leaving without treatment use the ED regularly as a way of accessing routine healthcare not otherwise accessible. It is unclear whether these patients received all necessary treatment, or whether their healthcare needs were met during their ED attendance.

The diagnosis that was most strongly associated with leaving without treatment was ‘nothing abnormal detected’. For these patients (3% of our sample), being recorded as leaving the ED without treatment may not be a concern as they may not have needed further treatment. However, patients with a primary diagnosis recorded as ‘not classifiable’ (17% of our sample), or with no diagnosis recorded (35%), were also relatively likely to leave without treatment. These patients may have left the ED without receiving potentially beneficial additional investigation or treatment.

Forty-nine percent of the study sample was recorded as not being registered with a general practice. We found that these patients had slightly greater odds of dying in the ED compared with patients recorded with a registered practice. This could be driven by poor access to routine healthcare or another factor correlated with general practice registration. For example, the homeless people in the poorest health may be the least likely to be registered with a general practice.^20^

Patients who received a psychiatric primary diagnosis (6% of our study sample) were more likely to be referred to another health care professional/provider than patients who received other diagnoses. This suggests that the ED is an important point of contact for PEH suffering from psychiatric conditions, enabling access to more specialized services. Some patients are likely to be suffering from acute psychiatric problems, in which case attending the ED is an appropriate way of gaining access to a specialized psychiatric service in an emergency. However, some PEH may use the ED as a point of contact for less urgent mental health issues. They may benefit from increased continuity of care through access to mental healthcare via other means, such as GP referral. Evidence suggests that often EDs are not well-equipped to deal with patients with long-term mental health problems.^34^

Patients who received a diagnosis of ‘social problems’ were highly likely to be discharged without follow-up. For these patients, the ED may be fulfilling a need that could be met more effectively and efficiently by other welfare services.

Descriptive features of the Type 1 ED attendances in this study are different to patients in the general population. Compared with a large sample of ED attendances in 2016/17,^25^ a greater percentage of patients in our study left before treatment (15% vs. 4%), and arrived by ambulance (56% vs. 30%). A lower percentage of attendances in this study resulted in admission to hospital (22% vs. 28%). A similar number of referrals were from emergency services (16% vs. 17% in our study), whereas a greater percentage of patients in our study were referred by the police (6% vs. 0.5%).

Compared to a study of 3271 attendances by PEH at a Type 1 ED in England,^24^ we found that a similar percentage of patients left without treatment (18% vs. 15% in our study). However, we found that a much lower percentage of patients died in the ED (1.2% vs. 0.2% in our study). The population served by that particular Type 1 ED may be in particularly poor health compared with the national average for PEH.

### Strengths and limitations

There are several strengths of this study. We used national hospital data for a long time period. This made it possible to have a large sample size with representation of patients from different groups, such as ethnic minorities and older patients who may not be well represented in smaller studies. The individual identifier also enabled us to access information relating to previous health care use during the 5 years before their ED attendance. The ED data we have used is administratively recorded so largely complete and consistent, and we do not rely on self-reported information regarding healthcare use.

There is no study of the validity of the no fixed abode flag used for this study. However, characteristics of the patients identified as PEH using this indicator and included in this study (mean age 38; 78% male; 13.5% of patients from ethnic group ‘White from any background other than British or Irish’, which is a large departure from the general population^35^) are similar to those reported in other studies of PEH in England.^20^^,^^36^^,^^37^

There are likely to be many ED attendances by PEH who provided a past address, or the address of a family member or a friend.^17^ We were unable to capture these attendances as they are indistinguishable from attendances by people who are genuinely housed. Homeless patients who are recorded with ‘no fixed abode’ may be more isolated than those who have an address to give. Patients also may be less likely to have residence coded as ‘no fixed abode’ if an address is seen to be relevant to their care, e.g. if they are admitted to hospital. These factors may affect generalizability if the no fixed abode flag systematically misses some homeless patients.

We were only able to analyze one diagnosis code related to substance misuse (‘poisoning’, which includes overdose). Given the high prevalence of substance use disorder in this population,^7^^,^^8^ we would have liked to include a diagnosis category for other (non-overdose) attendances relating to substance misuse. However, we were unable to identify these attendances from the diagnosis coding system used in the EDs included in this study.

### Implications for policy and research

Some PEH may use the ED as a point of access for routine health care and welfare support. This may be indicative of issues in accessing primary care and welfare services through other routes. This warrants further investigation.

Further research with PEH and health care providers is needed to investigate some of the findings in this study, e.g. the reasons why some patients are especially likely to leave without treatment. Further research is needed to understand care pathways for PEH and how they may be improved.

## Supplementary Material

ckac191_Supplementary_Data

## Data Availability

The Hospital Episode Statistics data that support the findings of this study are available from NHS Digital. Restrictions apply to the availability of these data. They were used under licence for this study, and so are not publicly available.

## Disclaimer

The funders were not involved in the design of the study, analyses, interpretation of the data, or the decision to submit results for publication.

Key points

- We analyzed the emergency department attendances of a large sample of people experiencing homelessness in England.
- Forty-nine percent of patients in the sample were recorded as not registered with a general practice.
- A high percentage of homeless patients leaving the ED without treatment may exacerbate unmet healthcare need in this population.
- Designing appropriate routes to access care and welfare services for people experiencing homelessness should be a priority.
