# Supplementary material for: Emergency department outcomes for patients experiencing homelessness in England: retrospective cross-sectional study
Source: Eur J Public Health. 2023 Jan 9;33(2):161–8. doi: 10.1093/eurpub/ckac191 (PMC10066478; doi:10.1093/eurpub/ckac191)
Supplement: ckac191_Supplementary_Data [file ckac191_supplementary_data.pdf]

SUPPLEMENTARY MATERIAL

Supplementary Figure 1: Flowchart of data preparation

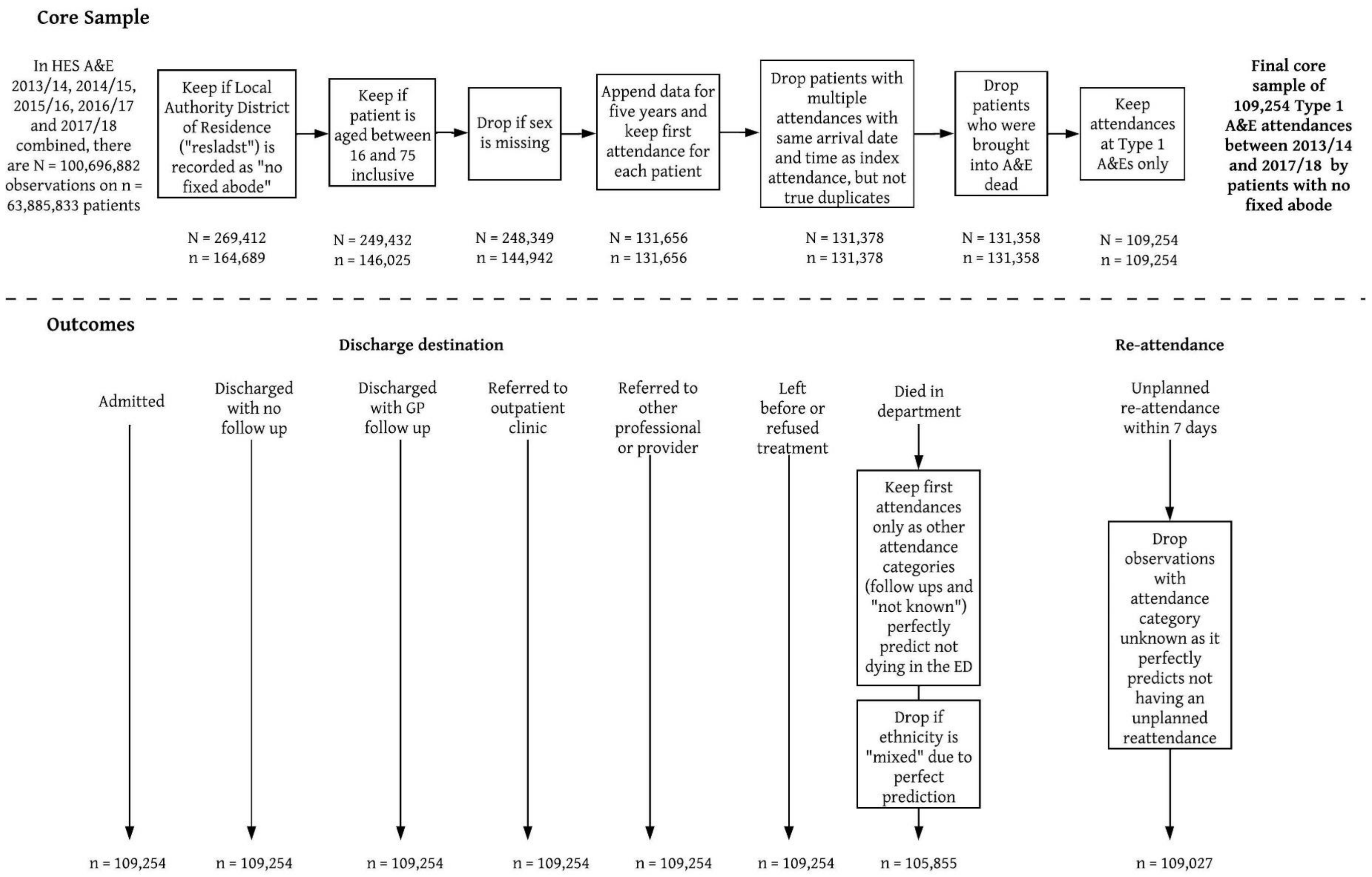

Supplementary Table 1: Original ethnicity categories and grouping for analysis

Ethnicity category in Hospital Episode Statistics	Frequency	%	Grouping
White British	47,712	43.67	White British or Irish
White Irish	1,489	1.36	White British or Irish
Any other White background	14,787	13.53	Any other White background
White and Black Caribbean (Mixed)	310	0.28	Mixed
White and Black African (Mixed)	131	0.12	Mixed
White and Asian (Mixed)	91	0.08	Mixed
Any other Mixed background	915	0.84	Mixed
Indian (Asian or Asian British)	1,253	1.15	Asian
Pakistani (Asian or Asian British)	646	0.59	Asian
Bangladeshi (Asian or Asian British)	272	0.25	Asian
Any other Asian background	1,794	1.64	Asian
Caribbean (Black or Black British)	835	0.76	Black
African (Black or Black British)	1,978	1.81	Black
Any other Black background	1,769	1.62	Black
Chinese	326	0.30	Asian
Any other ethnic group	8,539	7.82	Other
Missing	26,407	24.17	Missing
Total	109,254	100	

Data source: Hospital Episode Statistics Accident and Emergency, Type 1 A&E attendances by patients with no fixed abode aged 16 to 75 between 1st April 2013 and 31st March 2018.

Supplementary Table 2: Percentage of valid A&E diagnosis codes that belong to each code group

Primary diagnosis group code †	%	Categorisation
Laceration (01)	4.33	Traumatic injury
Contusion/abrasion (02)	2.47	Traumatic injury
Soft tissue inflammation (03)	2.01	Traumatic injury
Head injury (04)	4.48	Traumatic injury
Dislocation/fracture/joint Injury/amputation (05)	3.46	Traumatic injury
Sprain/ligament injury (06)	2.21	Traumatic injury
Muscle/tendon injury (07)	1.35	Traumatic injury
Nerve injury (08)	0.12	Traumatic injury
Vascular injury (09)	0.07	Traumatic injury
Burns and scalds (10)	0.40	Traumatic injury
Electric shock (11)	0.02	Traumatic injury
Foreign body (12)	0.40	Traumatic injury
Bites/stings (13)	0.32	Traumatic injury
Poisoning (inc. overdose) (14)	14.73	Poisoning
Near drowning (15)	0.01	Traumatic injury
Visceral injury (16)	0.08	Traumatic injury
Infectious disease (17)	0.91	Physical condition/disease
Local infection (18)	1.69	Physical condition/disease
Septicaemia (19)	0.21	Physical condition/disease
Cardiac conditions (20)	2.42	Physical condition/disease
Cerebro-vascular conditions (21)	0.38	Physical condition/disease
Other vascular conditions (22)	0.46	Physical condition/disease
Haematological conditions (23)	0.19	Physical condition/disease
Central nervous system conditions (exc. Strokes) (24)	2.72	Physical condition/disease
Respiratory conditions (25)	2.20	Physical condition/disease
Gastrointestinal conditions (26)	3.92	Physical condition/disease
Urological conditions (inc. cystitis) (27)	1.29	Physical condition/disease
Obstetric conditions (28)	0.14	Physical condition/disease
Gynaecological conditions (29)	0.47	Physical condition/disease
Diabetes and other endocrinological conditions (30)	0.47	Physical condition/disease
Dermatological conditions (31)	0.49	Physical condition/disease
Allergy (inc. anaphylaxis) (32)	0.24	Physical condition/disease

Facio-maxillary conditions (33)	0.46	Physical condition/disease
ENT conditions (34)	0.87	Physical condition/disease
Psychiatric conditions (35)	9.21	Psychiatric condition
Ophthalmological conditions(36)	0.94	Physical condition/disease
Social problems (includes chronic alcoholism and homelessness) (37)	3.27	Social problems
Diagnosis not classifiable (38)	25.97	Not classifiable
Nothing abnormal detected (39)	4.63	Nothing abnormal detected
	N = 70,708	

Data source: Hospital Episode Statistics Accident and Emergency, Type 1 A&E attendances by patients with no fixed abode aged 16 to 75 between 1st April 2013 and 31st March 2018.

† NHS Accident and Emergency diagnosis code groups.

Supplementary Table 3: Codes used to identify patients not registered at a general practice

Code	Description
V81997	Should be used when a patient presents, who is not currently registered at a GP practice, *but is eligible to be registered should they wish to*
V81998	Should be used where a patient should not have a registered GP practice
V81999	Should be used where it is not possible to determine a patient's registered GP practice code, but it is known that they should have one, or where it is impossible to determine whether they should or shouldn't have a registered practice (for instance the patient cannot communicate and is unidentified)

Source: NHS Digital, "NHS data model and dictionary: general medical practice code (patient registration)", https://www.datadictionary.nhs.uk/data_elements/general_medical_practice_code__patient_registration_.html

Supplementary Table 4: Distribution of counts of ED attendances, inpatient spells and outpatient appointments for patients with non-zero use

		Percentile									
	Min	10	20	30	40	50	60	70	80	90	Max
ED attendances	1	1	2	3	4	5	7	10	15	25	1,037
Inpatient spells	1	1	1	2	2	3	4	5	7	12	713
Outpatient appointments	1	1	2	4	5	7	10	14	20	33	2,254

Abbreviations: ED, emergency department.

Data source: Hospital Episode Statistics Accident and Emergency, Admitted Patient Care, and Outpatient datasets.

Supplementary Table 5: Definitions of indicators for zero, low, moderate, and high intensity of past health care use

Intensity of use	Definition	ED attendances	Inpatient spells	Outpatient appointments
No use	Count of zero	0	0	0
Low	Between count of 1 and 40th percentile	1 to 4	1 to 2	1 to 5
Moderate	41st to 80th percentile	5 to 15	3 to 7	6 to 20
High	>80th percentile	>15	>7	>20

Abbreviations: ED, emergency department.

Supplementary Table 6: Additional descriptive statistics: A&E attendance characteristics

	%
Attendance category	
First attendance	98.2
Planned follow up	0.2
Unplanned follow up	1.4
Not known	0.2
Referral source	
General medical practitioner	1.1
Self-referral	49.0
Local authority social services	0.1
Emergency services	19.4
Work	0.2
Educational establishment	0.1
Police	7.2
Health care provider	2.6
Other	19.2
General dental practitioner	<0.1
Community dental service	<0.1
Not known	1.2
Arrival mode	
Ambulance (inc. helicopter/air ambulance)	56.3
Other	43.2
Not known	0.5
Reason for attendance	
Road traffic accident	1.0
Assault	3.5
Deliberate self-harm	3.5
Sports injury	0.3
Firework injury	<0.1
Other accident	13.3
Other than above	77.0
Not known	1.4
n	54,173

Data source: Hospital Episode Statistics Accident and Emergency, Type 1 A&E attendances by patients with no fixed abode aged 16 to 75 between 1st April 2013 and 31st March 2018.

Supplementary Table 7: Regression results continued, control variables

	Admitted	Discharged with no follow up	Discharged with GP follow up	Referred to outpatient clinic	Referred to other professional or provider	Left without treatment	Died in department	Unplanned re-attendance within seven days
	(1)	(2)	(3)	(4)	(5)	(6)	(7)	(8)
Sex								
Female	0.89***	1.05**	1.16***	1.10*	1.15***	0.82***	0.82	0.76***
	[0.86,0.92]	[1.01,1.08]	[1.12,1.21]	[1.00,1.21]	[1.06,1.25]	[0.78,0.85]	[0.58,1.14]	[0.67,0.86]
Age								
16 to 20								
21 to 25	1.05	0.90***	1.01	1.17	1.12	1.04	0.33*	1.17
	[0.97,1.12]	[0.85,0.95]	[0.94,1.09]	[0.99,1.39]	[0.97,1.30]	[0.96,1.13]	[0.14,0.78]	[0.93,1.46]
26 to 30	1.14***	0.89***	0.93*	1.11	1.05	1.14**	0.62	1.17
	[1.06,1.22]	[0.84,0.94]	[0.86,0.99]	[0.94,1.32]	[0.91,1.22]	[1.05,1.23]	[0.30,1.26]	[0.93,1.46]
31 to 35	1.28***	0.80***	0.98	1.06	0.93	1.20***	0.55	1.37**
	[1.20,1.38]	[0.76,0.85]	[0.91,1.05]	[0.89,1.26]	[0.80,1.09]	[1.11,1.30]	[0.26,1.17]	[1.10,1.72]
36 to 40	1.37***	0.76***	0.93*	0.95	0.96	1.27***	0.83	1.36**
	[1.27,1.47]	[0.71,0.80]	[0.86,1.00]	[0.80,1.14]	[0.82,1.12]	[1.17,1.38]	[0.41,1.66]	[1.09,1.71]
41 to 45	1.46***	0.75***	0.93*	0.98	0.82*	1.25***	0.64	1.38**
	[1.36,1.57]	[0.71,0.79]	[0.86,1.00]	[0.82,1.18]	[0.69,0.97]	[1.15,1.36]	[0.30,1.38]	[1.10,1.73]
46 to 50	1.49***	0.75***	0.98	0.86	0.94	1.14**	1.18	1.23
	[1.39,1.61]	[0.71,0.80]	[0.91,1.05]	[0.71,1.04]	[0.80,1.11]	[1.04,1.24]	[0.60,2.33]	[0.97,1.56]
51 to 55	1.64***	0.73***	0.99	0.88	0.77**	1.06	1.81	0.95
	[1.52,1.77]	[0.68,0.78]	[0.91,1.07]	[0.72,1.08]	[0.64,0.93]	[0.97,1.17]	[0.93,3.50]	[0.73,1.25]
56 to 60	1.57***	0.73***	1.01	0.67**	0.81	1.05	2.43*	1.30
	[1.44,1.71]	[0.67,0.78]	[0.92,1.11]	[0.52,0.87]	[0.65,1.00]	[0.94,1.17]	[1.23,4.78]	[0.96,1.74]
61 to 65	1.78***	0.71***	1.12*	0.86	0.75*	0.75***	3.20**	1.21
	[1.61,1.96]	[0.65,0.77]	[1.01,1.25]	[0.65,1.14]	[0.58,0.98]	[0.66,0.86]	[1.60,6.41]	[0.84,1.75]
66 to 70	2.06***	0.72***	1.03	0.81	0.71*	0.56***	4.06***	1.33

	[1.85,2.29]	[0.65,0.80]	[0.92,1.17]	[0.59,1.10]	[0.52,0.97]	[0.47,0.66]	[1.98,8.29]	[0.87,2.05]
71 to 75	2.58***	0.65***	0.97	0.81	0.79	0.35***	7.77***	0.74
	[2.26,2.93]	[0.57,0.74]	[0.83,1.13]	[0.54,1.19]	[0.53,1.16]	[0.27,0.46]	[3.86,15.63]	[0.37,1.47]
Ethnicity								
White British or Irish								
Any other white background	1.00	1.00	1.19***	1.13	1.07	0.76***	0.69	0.66***
	[0.95,1.05]	[0.96,1.04]	[1.13,1.26]	[1.00,1.29]	[0.95,1.21]	[0.72,0.80]	[0.39,1.21]	[0.53,0.83]
Mixed	0.91	1.11	1.13	1.15	1.27	0.69***		1.33
	[0.79,1.04]	[1.00,1.24]	[0.99,1.30]	[0.84,1.58]	[0.97,1.68]	[0.59,0.82]		[0.92,1.92]
Asian	0.88**	1.10**	1.27***	1.06	0.95	0.76***	1.52	0.78
	[0.81,0.95]	[1.03,1.18]	[1.17,1.38]	[0.86,1.30]	[0.78,1.15]	[0.69,0.83]	[0.82,2.84]	[0.57,1.06]
Black	0.87***	1.11**	1.22***	1.03	1.41***	0.65***	1.55	0.81
	[0.81,0.94]	[1.04,1.19]	[1.13,1.32]	[0.84,1.25]	[1.21,1.64]	[0.59,0.71]	[0.80,2.99]	[0.62,1.06]
Other	1.09**	0.99	1.18***	1.20*	1.24**	0.67***	1.20	0.46***
	[1.03,1.16]	[0.94,1.05]	[1.11,1.26]	[1.03,1.39]	[1.09,1.42]	[0.62,0.72]	[0.70,2.07]	[0.32,0.65]
Missing	1.06**	0.99	1.06**	0.95	1.18***	0.88***	2.00***	0.68***
	[1.01,1.10]	[0.95,1.02]	[1.02,1.11]	[0.85,1.06]	[1.07,1.29]	[0.84,0.92]	[1.44,2.78]	[0.58,0.79]
Attendance Category								
First attendance	1.00	1.00	1.00	1.00	1.00	1.00		1.00
	[1.00,1.00]	[1.00,1.00]	[1.00,1.00]	[1.00,1.00]	[1.00,1.00]	[1.00,1.00]		[1.00,1.00]
Planned follow up	0.67*	1.14	0.96	2.41**	1.41	0.61*		1.76
	[0.47,0.96]	[0.87,1.49]	[0.68,1.35]	[1.37,4.24]	[0.77,2.61]	[0.39,0.96]		[0.66,4.71]
Unplanned follow up	0.76***	1.15**	0.82**	1.26	0.96	1.22**		3.15***
	[0.66,0.88]	[1.04,1.28]	[0.71,0.95]	[0.92,1.73]	[0.71,1.28]	[1.07,1.39]		[2.33,4.27]
Not known	1.65**	0.56**	0.26***	1.81	0.64	0.37***		
	[1.22,2.22]	[0.40,0.79]	[0.14,0.47]	[0.95,3.43]	[0.26,1.56]	[0.23,0.60]		
N	109,254	109,254	109,254	109,254	109,254	109,254	105,855	109,027

95% confidence intervals in brackets

* $p<0.05$, ** $p<0.01$, *** $p<0.001$

Data source: Hospital Episode Statistics Accident and Emergency attendances by patients with no fixed abode aged 16 to 75 between 1st April 2013 and 31st March 2018.

All models estimated using logistic regression. Coefficients show odds ratios.

Sample includes attendances at Type 1 EDs only. Models additionally include four binary indicators for whether the attendance is categorised as a first attendance, planned follow up, unplanned follow up or unknown.

Due to small number of patients who died in the department, diagnosis indicators were not included in model 7 due to perfect prediction. Model 7 also includes first attendances only due to perfect prediction. Patients with Mixed ethnicity were removed from the sample due to perfect prediction.
